# Practical interpretation of ILCOR and neonatal resuscitation program recommendations for initial oxygen concentration for neonatal resuscitation/stabilization

**DOI:** 10.1038/s41372-026-02608-x

**Published:** 2026-03-13

**Authors:** Satyan Lakshminrusimha, Deepika Sankaran, Christina Sollinger, Payam Vali, Evan Giusto

**Affiliations:** 1 Department of Pediatrics, University of California at Davis Children’s Hospital, Sacramento, CA USA; 2 Patient Care Services Advanced Practice: Neonatal, University of California at Davis Health, Sacramento, CA USA

**Keywords:** Respiration, Metabolism

The initial inspired oxygen concentration for resuscitation and stabilization of term and preterm neonates has changed over the years. While previous versions of recommendations from the International Liaison Committee on Resuscitation (ILCOR) and American Heart Association (AHA) and American Academy of Pediatrics (AAP) Neonatal Resuscitation Program (NRP) guidelines had specific initial oxygen (O_2_) concentrations (i.e., 100% O_2_ for term and preterm in 2000) [1] or narrow ranges (i.e., 21% for term and 21-30% for preterm in 2015) [2], the current suggestion is that it is reasonable to use ≥ 30% O_2_ for preterm infants < 32 weeks (weak recommendation, low-certainty evidence) [3, 4]. However, this evolving guidance has made it challenging to establish clear and consistent protocols for managing oxygenation during the stabilization of preterm infants in the delivery room [3, 4].

Studies in preterm infants <32 weeks have shown that failure to achieve a preductal SpO₂ of ≥80% within the first five minutes after birth is associated with a poor prognosis, including higher rates of mortality and morbidity such as severe intraventricular hemorrhage [5, 6]. Inability to achieve an SpO_2_ of ≥80% by 5 min may result from two main factors. The first is iatrogenic (providing an inadequate inspired oxygen concentration (FiO_2_) or positive pressure, a poor interface such as a mask leak, or targeting lower than physiological SpO_2_ targets [7]). Some of these factors are being currently investigated in clinical trials (OptiSTART trial, NCT05849077) and the recently published Torpido 3060 trial [8]. The second category includes neonatal factors such as extreme prematurity, severe lung disease, lack of antenatal steroid exposure, and cardiac dysfunction, where target SpO_2_ is not achieved despite providing optimal or maximal FiO_2_ and pressure.

**Fig. 1:**
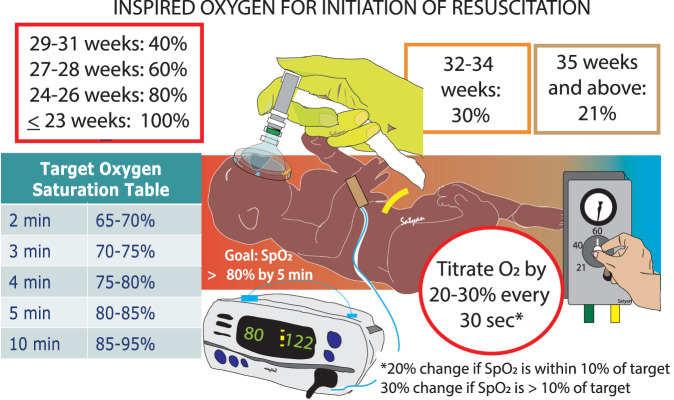
Suggested initial oxygen saturations for collecting prospective data in preterm infants < 32 weeks’ gestational age. The current edition of the Textbook of Neonatal Resuscitation (9^th^ edition) states that it is reasonable to use 30–100% oxygen for initial resuscitation/stabilization of preterm infants < 32 weeks gestation. The approach outlined in this figure suggests higher initial inspired oxygen for more preterm infants but still within the 30–100% range considered reasonable by ILCOR and NRP.

We believe that all preterm infants <32 weeks of gestation at birth should not have the same initial FiO_2_ for resuscitation. A study conducted by the California Perinatal Quality Care Collaborative (CPQCC) demonstrated that starting with ~30% O_2_ (and titrating based on SpO_2_), 70.3% of infants born at ≤23 weeks, 50% of those born at 24-26 weeks, and 38.5% of those at 27–28 weeks had a 5-min SpO_2_ of <80% [6]. Based on these data, we propose using different initial O_2_ concentrations for the resuscitation of preterm infants, while continuing to prospectively collect data on heart rate, preductal SpO₂, and associated morbidities and mortality (Fig. 1).

For initial inspired O_2_ for resuscitation and stabilization of preterm infants, we urge individual institutions create their own algorithms within the confines of ILCOR suggested range of 30–100% (O_2_). A sliding scale based on estimated gestational age at birth being adopted at our institution is shown below (Fig. 1):

- 40% oxygen for 29 0/7 to 31 6/7 weeks
- 60% oxygen for 27 0/7 to 28 6/7 weeks
- 80% oxygen for 24 0/7 to 26 6/7 weeks
- 100% oxygen for ≤ 23 6/7 weeks
  Subsequent titration of inspired O_2_ is an essential component of achieving the target SpO_2_ of ≥80% by 5 min. The Textbook of Neonatal Resuscitation (9^th^ edition) states that “a reasonable approach is to adjust the FiO_2_ in increments of 20 to 30% every 30 s until the oxygen saturation target is achieved.” [4] Based on limited data from animal studies [9], and understanding that apnea and vocal cord closure [10, 11] may influence oxygenation in preterm infants being resuscitated with a mask, we suggest titrating inspired O_2_ every 30 seconds based on the difference between the observed SpO_2_ and target SpO_2_:
  - By 20% if SpO_2_ is <10% outside the range (e.g., target SpO_2_ lower limit is 70%, and infant’s preductal SpO_2_ is 61%, then increase O_2_ by 20%)
  - By 30% if SpO_2_ > 10% outside the range (e.g., target SpO_2_ lower limit is 70%, infant’s preductal SpO_2_ 50%, then increase O_2_ by 30%)

We recognize that clinical trial data do not yet support these guidelines. During non-invasive ventilation, delays between adjustment of oxygen blender and the actual concentration reaching the face mask can occur. In nearly half of clinical observations, the intended FiO_2_ was not achieved before the next titration [12]. Optimal titration methods remain an important research gap in neonatal resuscitation.

One of the key factors necessary to establish the lungs as the primary site of gas exchange is ventilation of the lungs [13], which results in an increase in pulmonary blood flow at birth [14]. Increased pulmonary blood flow is enabled by increased right ventricular output due to pulmonary vasodilation and increased left-to-right ductal shunt. Based on animal data, initiating resuscitation with 21% O_2_ in term lambs appears to decrease pulmonary arterial pressure (PAP) and create a gradient between aortic pressure and PAP, thereby reversing the ductal shunt to left-to-right and increasing pulmonary blood flow [15]. However, initiation of resuscitation with 21% O_2_ in preterm and rapid titration does not result in an adequate decrease in PAP. In contrast, initiating resuscitation in preterm lambs with 100% O_2_ significantly decreases PAP as compared to systemic arterial pressure leading to reversal of ductal flow [15]. In addition, initial resuscitation with 100% O_2_ may prevent hypoxemia and potentially stimulate glottic opening and breathing effort [10, 16].

Initiating stabilization of preterm infants < 30 weeks gestation at birth with 100% O_2_ improved breathing effort and oxygenation, and shortened the duration of mask ventilation compared to 30% O_2_, without increasing the risk for hyperoxia or oxidative stress in a randomized controlled trial (RCT) by Dekker et al. [17]. Recently, Katheria et al. compared 100% vs. 30% O_2_ for 90 s during deferred cord clamping followed by 30% oxygen in preterm infants at 22–28 weeks gestational age [18]. Preterm infants in the 100% O_2_ group had increased chance of achieving SpO_2_ ≥ 80% by 5 min with higher median SpO_2_ by 5 min. Although FiO_2_ over the first 10 minutes after birth was similar between the two groups, the median SpO_2_ was significantly different at 5 min between the two groups (88% vs. 69% in the 100% and 30% initial O_2_ groups, respectively). These two small studies suggest potential respiratory and oxygenation benefits of initiating resuscitation in extremely preterm infants with 100% O_2_. The NETMOTION meta-analysis did not include the DOXIE trial and showed with low certainty, a lower mortality with the use of high (90–100%) oxygen compared to low oxygen (≤ 30%) among <32 weeks infants [19].

With this background, it is clear that extremely preterm infants have a unique physiology that differentiates them from term infants [20]. A universal optimal approach for initial FiO₂ in infants <32 weeks is unlikely. Therefore, ongoing surveillance of outcomes and therapies is essential to evaluate the effects of oxygen exposure.

With ILCOR and NRP recommending 30–100% O_2_ at initiation of resuscitation and stabilization of preterm infants <32 weeks’ gestation, it is up to each medical center to develop appropriate guidelines and commit to titration of oxygen once the heart rate and SpO_2_ are available. While short-term outcomes appear favorable with higher initial FiO₂, the potential long-term effects of early hyperoxic exposure remain insufficiently understood, underscoring the need for continued surveillance and prospective studies. Prospective collection and pooling of data on 5-minute preductal SpO_2_, heart rate, major morbidities and mortality using a sliding scale as shown in Fig. 1 while awaiting outcomes from further trials, are as critical as conducting RCTs in determining the optimal approach to initial FiO_2_ for extremely preterm infants. These fragile, preterm infants cannot wait another decade for a trickle of non-committal knowledge. Novel trial methods such as adaptive platform trials [21] or use of large registries evaluating protocols based on physiology are needed to quickly collect evidence that includes measures of oxidative stress for an initial FiO_2_ strategy for resuscitation of preterm infants.
